# Simulated Environmental Conditioning of PHB Composites Reinforced with Barley Fibres to Determine the Viability of Their Use as Plastics for the Agriculture Sector

**DOI:** 10.3390/polym15030579

**Published:** 2023-01-22

**Authors:** Helena Oliver-Ortega, Fernando Julián, Francesc Xavier Espinach, José Alberto Méndez

**Affiliations:** 1 Department of Materials Science and Engineering, Universitat Politècnica de Catalunya, Colom 1, 08222 Terrassa, Spain; 2 LEPAMAP-PRODIS Research Group, University of Girona, EPS-Ed.I, Maria Aurèlia Capmany 61, 17003 Girona, Spain

**Keywords:** polyhydroxybutyrate (PHB), barley fibres, biodegradable composites, simulated environmental characterization, mechanical properties

## Abstract

Nowadays, the search for new materials with a sustainable character to reduce the production of residues is under continuous research. In this sense, fully biodegradable composites based on polyhydroxybutyrate and different pretreated fibres coming from barley straw have been fabricated, and their resistance to environmental controlled conditions have been characterized. The materials were already compounded in a kinetic mixer and injection-moulded as specimens for tensile assay to be aged in a Xenotest chamber so as to simulate environmental conditioning. The samples, after accelerated aging, were characterized thus: mechanical characterization (tensile assay), water uptake (immersion and contact angle), and surface observation (optical and SEM microscopy). The incorporation of the fibres helps the composite to keep its structure for a longer time. On the other hand, the presence of the fibres increases the water uptake capacity to allow water permeation in the composite, which allows final degradation, characterised by a significant drop in properties after one month of exposure to simulated environmental conditions.

## 1. Introduction

Beyond considering the elimination of polymeric materials from the retail market, domestic use, and packaging components, the current situation for polymeric materials, as well as composites based on polymers as continuous phase, is experiencing a continuous growth due to their use of sustainable/biodegradable materials, expecting an enormous interest for the next decades. There are estimates of the use of biodegradable polymers, as substitutes of petroleum-derivative matrices, which suggest an increase its use three to four times their current use in 2022 (source: Precedence Research report). During 2021 the market of biodegradable polymers at the global level was close to 11.2 billion USD and its expectation of use by 2030 is estimated at 46.2 billion USD, representing a 400% increase.

The main representatives of this family of polymers are headed by polyesters such as polylactic [1,2,3] acid, thermoplastic starch [4,5], and polyhydroxyalcanoates [6,7]. In fact, these polymer matrices are already the most often used biopolymers at the moment due to their intrinsic properties, such as moderate mechanical performance, low densities (comparable to those derived from petroleum), and easily processable, using the same methodologies as conventional polymers and composites, but adding a new paradigm in its use: biodegradability. Thus, its use does not damage environmental policies surrounding residues, but rather helps their elimination from the environment after its use, as CO_2_ and H_2_O, or as oligomers which are easily degradable.

This interesting family of materials also has limitations derived from their higher production price, which is decreasing continuously, but is still high compared to petroleum-derived matrices. 

In our socio-economic context, the effort for their promotion is guided towards decreasing their production price, and one of the alternatives is their combination with third components that allow for the keeping of their mechanical performance for a specific period, as well as keeping the biodegradable character. This concept is focused on the fabrication of composites based on biodegradable/sustainable polymeric matrices, which are reinforced/loaded with cellulosic fibres. In this sense, different kinds of cellulosic fibres are suitable for such an end: wood-derived fibres [8,9,10], plant stalks [11,12], cellulosic processed industrial residues [13,14,15], and agricultural residues [16]. The use of one of these other reinforcements depends on the expected final properties as well as the desired price of the raw materials. The residues derived from annual plants, such as cereals, or even the felling of trees [17] are the most economically competitive. For more than 20 years, such reinforcements and loads have caught the interest of different research groups around the world, but the current socio-economic situation has produced an increase in this interest.

The combination of both components, a biodegradable polymer matrix and cellulosic reinforcements, allows the obtainment of composites for applications in short-term performance, helping in the development of policies of environmental control in a more economically competitive manner [18]. In this sense, the use of residues of cellulose or plants without added value [16,19,20,21,22], such as barley straw [23,24], can ensure the provisioning of raw materials at a very low price, so as to guarantee a continuous production, therefore decreasing the cost over a longer time. Moreover, the use of residues can also help to implement policies of rural development due to the added value reached by the residue. The incorporation of agricultural residue into the polymer must permit the decomposition process of the plain matrix, or even improve it [25], so as to use its biodegradable character. 

Using the above arguments, this paper has planned an approach to produce composites to be used in the agricultural sector as one-use materials with a high capacity to be degraded in the field, so as to avoid their elimination after use. That is the case for packaging plastics for plants, covers for grass, and plant pots, which after use could be degraded by the environment, but retain their structure at least until the end of their use. PHB has been considered as a potential polymer matrix to be loaded with barley residual fibres, collected from the Spanish-French border, according to the BIOPLAST project financed by the Interreg-POCTEFA program. The composite will be fabricated by a semi-industrial batch technology, and will be submitted to an environmental simulation conditioning, with subsequent characterisation (morphological, mechanical, and water uptake) to determine their potential use in this sector.

## 2. Materials and Methods

### 2.1. Materials

PHB polymer matrix (degree P209) was acquired from BiomerTM (Schwalbach, Germany). Barley straw, a by-product from barley crops, was obtained from farmers in the area between the Spanish-French border, as established by the financer (Interreg-Poctefa program 2014–2020). The fibres were processed and/or chemically treated using different methods: mechanical milling (sawdust, SD); mechanical defibration with a water assisted Sprout-Waldron equipment (sprout, SP); chemical delignification with a NaOH cooking process (cooked, CK); and finally, a bleaching process (bleached, BL). 

Hydrogen peroxide (30 %(*w*/*w*) in water, Scharlau, Sentmenat, Barcelona, Spain) and sodium hydroxide in reagent grade (purity 97%, Scharlau, Sentmenat, Barcelona, Spain) were used to perform the chemical treatments of the fibres before incorporation into the polymer matrix.

### 2.2. Methods

#### 2.2.1. Composite Compounding

All the materials were compounded in a Gelimat kinetic mixer (Draiswerke, Ramsey, NJ, USA). The polymer and the different pre-processed barley fibres, at specific compositions from 10 to 30 wt%, were added through the feeder, setting an initially low mixing rate of the engine (300 rpm), which was then increased up to 2500 rpm to achieve the melting of the polymer matrix, finally producing the consolidation of the composite. The code of each formulation includes the kind of fibres used as reinforcement (SD, SP, CK, and BL) and its composition in the composite (e.g., SD-10). After the consolidation, the material was discharged, cooled down, and pelletized to obtain an optimal particle size capable to be injection-moulded. This pelletization process was carried on in a blade mill.

#### 2.2.2. Sample Processing

The specimens of each material were processed by injection moulding using an Aurburg 220M 350-90 U injection machine (Aurburg, Loßburg, Germany). The temperature profile was 165-165-165-158 °C and the pressures ranged between 300–400 bar depending on the fibre content.

#### 2.2.3. Sample Conditioning Prior Characterisation

The samples were stabilized in a climatic chamber (Dycometal, Sant Boi de Llobregat, Spain) in terms of temperature and relative humidity according to ASTM D618: 23 °C ± 1 °C, 50% RH for 48h.

#### 2.2.4. Simulated Environmental Conditioning

A total of 10 specimens of each injection-moulded formulation were submitted to a simulated environmental assay using a Xenotest Alpha chamber (Atlas/Ametek, Mount Prospect, IL, USA), equipped with a xenon light emitter and humidity controller up to 99% RH. The equipment also includes a deposit of distilled water to generate humidity. According to ISO 4892 standard specification, an environmental simulation routine of the assay was designed. The different stages and conditions of the procedure are summarized in Table 1.

The exposure time was reached after cyclic application of the routine for 1, 2 and 4 weeks.

#### 2.2.5. Optical Observation of the Samples

The surface of the samples after exposure to radiation, temperature, and humidity in the Xenotest chamber was observed by means of macroscopic photography using a Canon Powershot G12 still camera, equipped with an objective of x5-optical magnification and a diaphragm overture of 2.8–4.5.

#### 2.2.6. Mechanical Characterisation

After different exposure periods under the simulated environmental conditions (1, 2, and 4 weeks), the samples were tested under tensile stresses to determine the effect of the environmental conditioning. The samples were characterised using a universal testing machine (IDMtest, Zamudio, Spain) equipped with a 5kN load cell. The characterisation was carried out at room temperature, obtaining the results for Young’s modulus (with extensometer), ultimate tensile strength, and elongation at break. Each property was calculated from at least five values from individual specimens, and the corresponding average and standard deviation were calculated according to ASTM D638.

#### 2.2.7. Water Contact Angle Measurements

The water contact angle of the surface of all the materials prepared in this work was evaluated after exposure to a controlled environment. A DSSA25 drop-shape analyzer from Krüss GmbH (Hamburg, Germany) was used. The samples were tested using the sessile water droplet method, determining the angle between the plane of the surface of the sample and the tangent of the drop of water deposited on it. The experiments were carried out at room temperature.

#### 2.2.8. Water Uptake Behaviour

A modification of the capacity for water absorption during the exposure to simulated environmental conditions ws expected. Therefore, water uptake behaviour was evaluated. Fragments of each material, using three specimens per formulation, were dried in an oven at 80 °C until reaching constant weight, and later were immersed in distilled water at 23 ± 1 °C. The samples were extracted from the hydration medium, weighed, and again immersed for a new period of immersion. The percentage of water uptake (WUptake%) was calculated using Equation (1):

$$\mathrm{WUptake}\%=\frac{m_{t}-m_{0}}{m_{0}}\times 100$$

where m_0_ and m_t_ are the weight of the sample before immersions and at a specific time of immersion).

#### 2.2.9. Morphological Study of the Fracture of Tensile Specimens

The surfaces of fracture of the samples—firstly submitted to environmental degradation and secondly tested under tensile stress—were characterised by means of scanning electron microscopy (SEM) (Zeiss DSM 960 microscope). Samples were sputter-coated with a nanometric layer of gold. The samples were observed in the area close to the edge of each sample to observe the behaviour of the environmental conditioning to the surface of the sample and just below it.

#### 2.2.10. Statistic Test

Significant differences of the results of all the properties studied in this work have been determined by means of a one-way ANOVA test. Variance of the averages of the values was evaluated in a confidence interval of 95% (*p* = 0.05) to verify statistical differences between the two populations of results.

## 3. Results and Discussion 

### 3.1. Surface Observation

**Figure 1:**
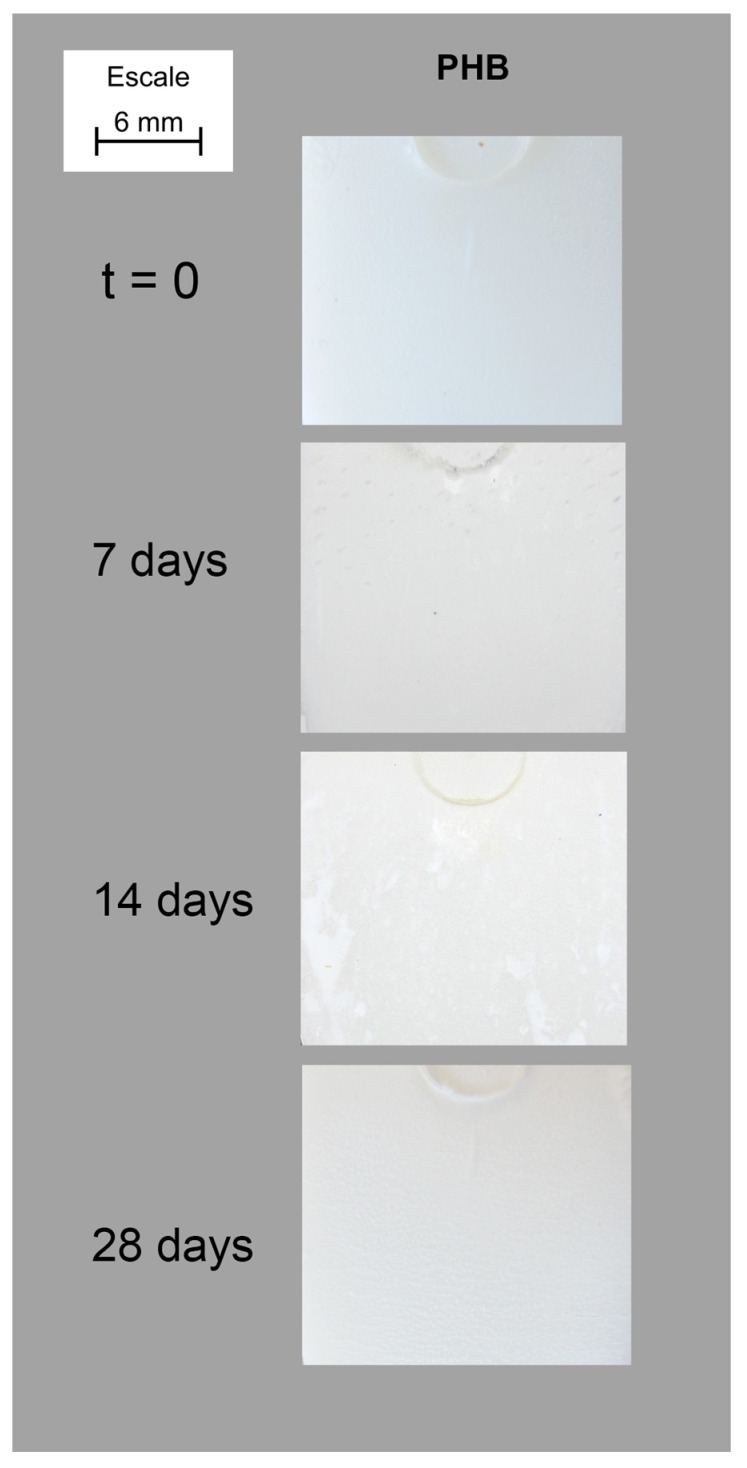
Evolution of the surface of plain-PHB samples inside Xenotest chamber.

Once the samples have been submitted to environmental conditioning, the surface of the samples of the composites was observed from macro pictures taken with a still camera. Figure 1 shows the behavior of plain PHB showing the permanence of the white colour of this matrix for the full period of exposure. However, a change in the whiteness of the samples is clearly appreciable. Moreover, some surface deposition could also be observed. This effect was more visually observable when fibres were added to the composite composition as an increase in the loss of colour was observed, independently of the fibres added.

**Figure 2:**
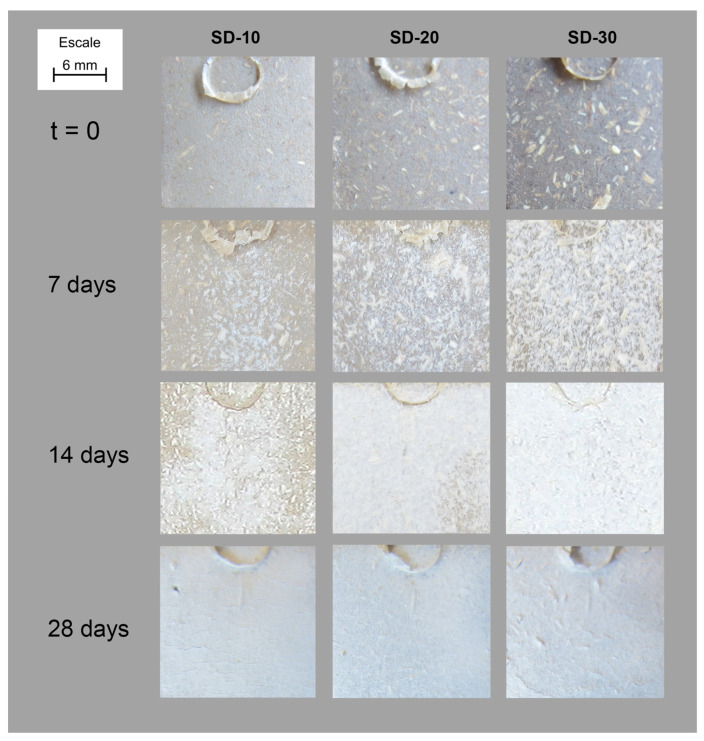
Evolution of the surface of SD-composites inside Xenotest chamber.

An example of this behaviour is Figure 2, where the evolution of the surface of SD-composite formulations (SD-10, SD-20 and SD-30) after 1, 2 and 4 weeks of exposure is represented. It is possible to observe the big granulometry of barley straw fibres, as only a milling process was performed to reduce their particle size to improve their incorporation into the matrix during mixing.

**Figure 3:**
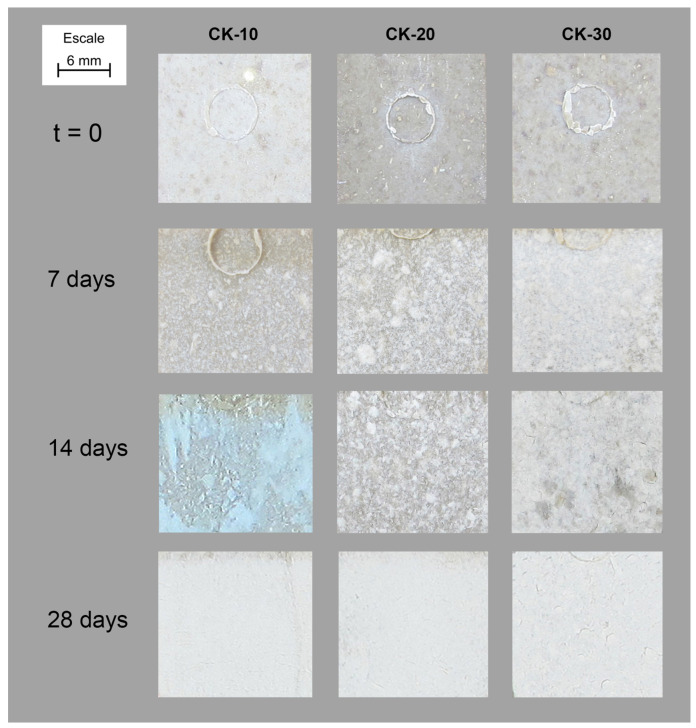
Evolution of the surface of CK-composites inside Xenotest chamber.

After 7 days a loss of colour was detected. Each picture also includes the footprint of the ejector (from the injection moulding processing) which allows the observation of the surface shading due to loss of material because of degradation under characterisation conditioning. Radiation, together with the humid atmosphere, produces bleaching of the material and degradation. This bleaching phenomenon is known as the polymer weathering effect based on a photo-oxidation process governed by the formation of radicals on the surface of the material due to outdoor radiation. These radicals react with atmospheric oxygen, which creates organic peroxides responsible for the degradation of the polymer chain [26]. Additionally, this bleaching effect is deeper as fibres content increases, with the SD-30 formulation the formulation that experienced a higher loss of colour. This effect was also observed in samples reinforced with mechanical defibrated (SP), cooked (CK) (Figure 3), and bleached fibres (BL). The differences observed are related to the different particle size of each reinforcement, with that of sawdust (SD) being the biggest and that of bleached fibres the smallest.

### 3.2. Water Contact Angle

**Figure 4:**
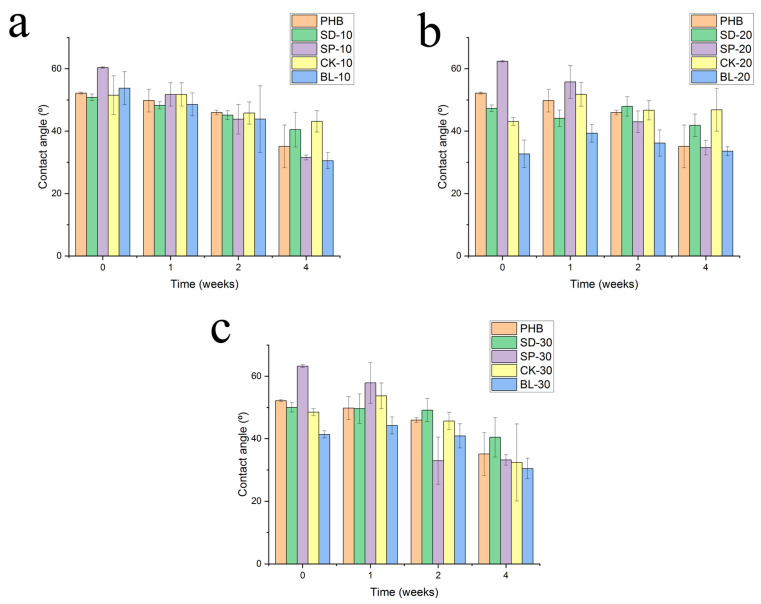
Evolution of the contact angle of composites with the exposure time on Xenotest. Reinforcement: (**a**) 10%, (**b**) 20% and (**c**) 30%.

It is expected that the bleaching phenomenon may be related to a change in the chemical structure of the materials on the surface. In this sense, an increase in polarity could be consistent with this effect, which is attributed to the oxidation of the surface [26]. A way to determine the change in the chemical structure of the surface of the materials is the determination of the contact angle of the materials. Figure 4 shows the values of the contact angle of the composites reinforced with all the processed fibres as well as the time of exposure under the environmental conditions.

In general terms, the exposure to this simulated environment induced a decrease in the contact angle of the surface of the materials, which was highest between week 2 and week 4. If reinforced with bleached fibres (BL) showed the lowest value of contact angle compared with the other composites at the same percentage of reinforcement and the same time of exposure. This phenomenon might be related to the high content of cellulose that this reinforcement has due to the bleaching process, which causes a decrease of the content in lignin. In this sense, an increase in cellulose ratio must be related to a higher polarity of the material due to the oxidation of the polar groups.

The presence of heterogeneous materials induces a high dispersion in the individual values of contact angle, conditioning, and—in some cases—high values of standard deviation. Analyzing the variance (ANOVA test), in different cases the obtained values for the same material at different periods of exposure time were not statistically different for consecutive periods of measurements, but were statistically different for nonconsecutive ones. That is the case of formulation BL-10. Average values of contact angle after 1, 2 and 4 weeks were 48.6, 43.8, and 30.5º, respectively. The values for week 1 and 2 were not significatively different due to the high value of standard deviation of the average at 2 weeks. The same trend was obtained for the value at 4 weeks when compared to that obtained for 2 weeks. However, when comparing week 1 and week 2, average values were statistically different.

### 3.3. Water Uptake Behaviour

**Figure 5:**
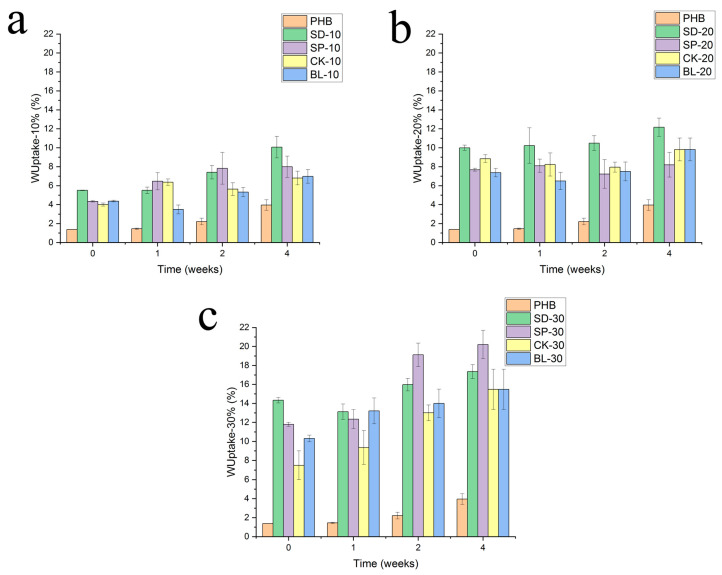
Evolution of the water uptake of composites with the exposure time on Xenotest. Reinforcement: (**a**) 10%, (**b**) 20% and (**c**) 30%.

This modification of the surface contact angle, together with the expected degradation of the material, can also influence the modification of the capacity for water uptake. Figure 5 shows the evolution of the water uptake capacity of the materials considering the kind of added fibre and exposure time under the environmental conditions. It is easy to observe that by increasing the exposure time to radiation, the capacity for water uptake was increased, with the formulation reinforced with 30wt% of fibres—independently of the kind of fibres added to the formulation—which shows the highest value of water uptake for every time. The presence of a higher content of fibres also helps the permeation of water through the material due to the already-known poor compatibility of lignocellulosic fibres and biodegradable polymers [27,28]. This phenomenon produces an increase of interfaces between both components of the composite, thereby creating ways for water diffusion through the material. In this sense, the increase in the capacity for water diffusion, like a porous structure, also may be interesting in considering the biodegradable character of the composite, and an increase in the capacity to interact with water can also accelerate the hydrolytic process of degradation of the material [29]. This is a key point in the fabrication of these composites so that the composite cannot harm the biodegradable character of the material.

Another interesting result can be observed in Figure 5, where water uptake is now shown by comparing all the fibres at the same composition. Considering the percentage of fibres added to the polymer matrix, the water uptake is generally higher for the formulation including fibres such as sawdust (SD formulations). Such a formulation is based on the incorporation of fibres obtained by a milling process of barley straw without any other prior processing, neither chemical or physical, which shows the highest particle size. This particle size also helps to create higher interfaces with low compatibility between the polymer and the fibres. Moreover, these fibres include every chemical component of the original lignocellulosic resource, thereby increasing its heterogeneity and consequently the incompatibility with the polymer matrix. On the other side, the formulations including cooked and bleached fibres show the lowest water uptake capacity for every composition (10, 20, and 30 wt%), which indicates that the homogeneity in the structure, due to a higher content in cellulose, helps compatibility by decreasing the capacity of water uptake, showing a more stable character against aqueous media. In this sense, it is expected that the formulations with sawdust and defibrated fibres can retain the biodegradable character when helped by a better diffusion of water through their structure.

Turning to the statistical study of the variance, considering the same material after different periods of time of exposure, consecutive values of the average of water uptake are significatively different for most formulations. In contrast to the study of the contact angle, the experiment of water uptake offers lower values of standard deviation, confirming the trends of evolution of the property. Only a few cases were not statistically different. That was the case of formulation SP-30 at 2 and 4 weeks of exposure, where the average values were not statistically different, although the evolution of the trend of the averages is similar to formulations where they were statistically different.

### 3.4. Mechanical Performance

**Figure 6:**
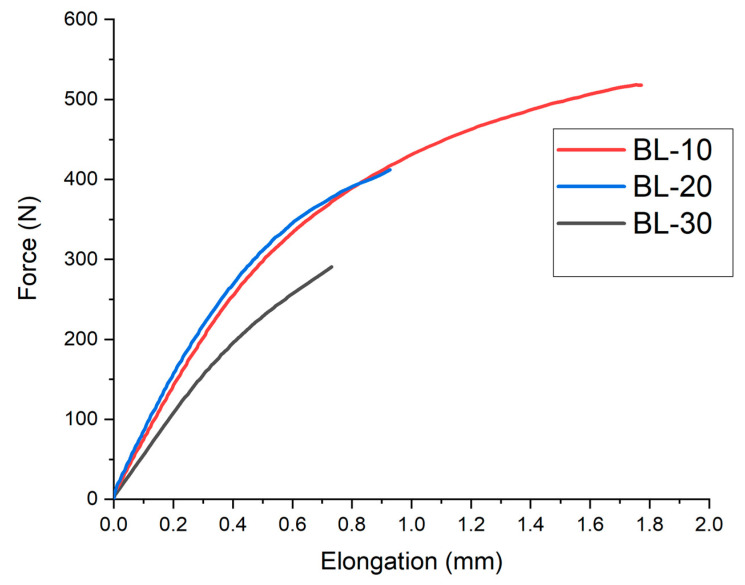
Force-elongation trends of composites of PHB reinforced with BL barley fibres tested after 14 days of exposure to environmental conditions.

A mechanical test under tensile stresses was performed, which obtained force-elongation profiles as showed in Figure 6. It is possible to observe that the obtained trends of composites are analogous to that of conventional non-reinforced polymeric materials, identifying the linear elastic trend (where Young’s Modulus is measured) as well as the change to non-linear trend, identified as the plastic evolution. The ultimate tensile strength was identified as the value of strength in the breaking point, and the elongation at break as the value of elongation to break when the sample was broken. 

**Figure 7:**
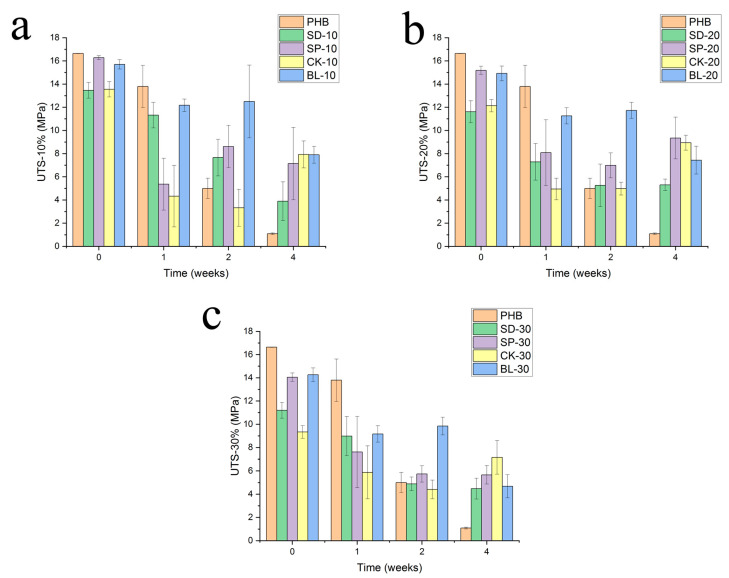
Evolution of the ultimate tensile strength of composites with the exposure time in Xenotest chamber. Reinforcement: (**a**) 10%, (**b**) 20% and (**c**) 30%.

According to this explanation, ultimate tensile strength has been determined and are summarized in Figure 7. The incorporation of fibres showed two different behaviours depending on the exposure time to the environmental conditioning. During the first 7 days, plain PHB showed higher mechanical strength than the composites reinforced with barley straw fibres. After this first week, the structure of plain-PHB samples showed a drastic decrease in mechanical strength but when reinforced with barley fibres this decrease stopped, keeping the structural strength for a longer time. Thus, after 28 days of exposure, the samples based on only PHB have a strength even lower than 1 MPa, while those reinforced with fibres ranged between 4–8 MPa depending on the pre-treatment of the fibre and its percentage in the composition.

In this sense, the incorporation of barley straw fibres improved the mechanical stability and integrity of the material for a longer time compared to the performance of the plain polymer matrix. The incorporation of fibres to improve the mechanical stability of polymer matrices has often been reported as cellulosic materials have longer degradation periods [30,31].

**Figure 8:**
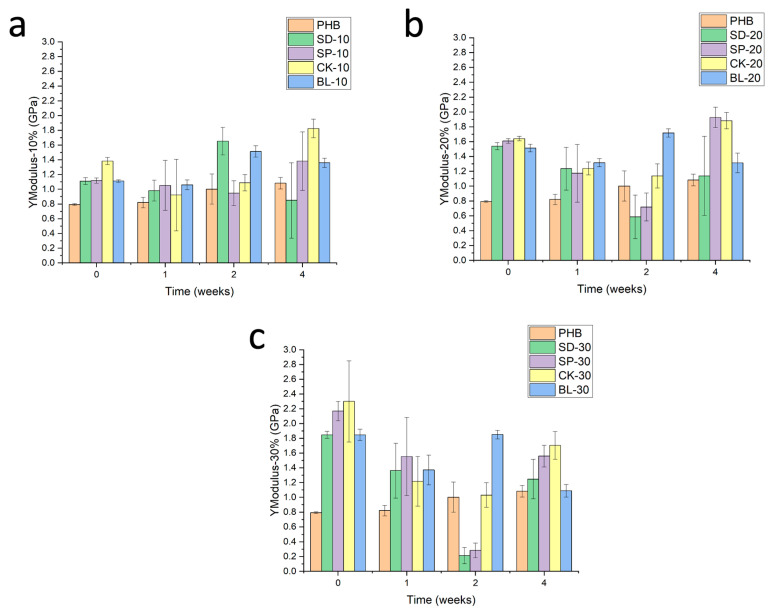
Evolution of the Young’s Modulus of composites with the exposure time in Xenotest chamber. Reinforcement: (**a**) 10%, (**b**) 20% and (**c**) 30%.

Young Modulus profile is shown in Figure 8. Young’s Modulus of plain PHB is very stable during the characterization period (4 weeks of exposure). In the case of the formulations reinforced with barley fibres, this behavior is more random due to the high heterogeneity of the materials related to the low compatibility fibre matrix. In general terms, two different behaviours have been observed when the composites are studied: composites reinforced with SD and SP fibres and that of CK and BL. The reinforcing of the composite by means of fibres with the same composition as the original barley fibres (SD-SP) simulates the behavior of PHB during the first stages of the exposure at low percentages of addition. After 2 weeks of exposure, the degradation of the materials involved a drastic decrease in Young’s modulus, which is higher when the composition of fibres in the composite increases. The low compatibility of the lignocellulosic residue together with the previously reported high capacity for water uptake induced by these fibres gave rise to a plasticization effect on the material due to the absorption of water. 

**Figure 9:**
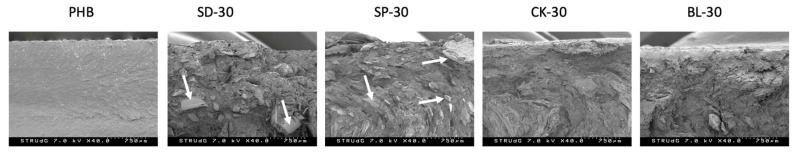
SEM observation of the breaking area of samples testes under tensile stresses. White arrows identify particles of the reinforcement.

Secondly, the incorporation of CK or BL fibres, with a higher composition of cellulose and a lower capacity to water absorption, led to keeping the stiffness of the composite for a longer time, showing a behavior closer to that of plain PHB for formulations of 30wt% of reinforcement after 2–4 weeks of exposure. In this sense, it is expected that the biodegradability of the formulations could be higher in the case of SD-SP fibres compared to that of CK-BL due to the higher permeability to water. As previously mentioned, this higher degradation of SD-SP-reinforced composites is related to their higher capacity to absorb water due to the low compatibility of the composite components. This low compatibility also can be confirmed by SEM observation of the breaking area of the specimens tested under tensile stresses. Figure 9 shows a higher heterogeneity of composites reinforced with SD or SP fibres, compared to those of CK and BL. This heterogeneity allows higher water permeation and consequently a higher plasticizing effect producing a higher decrease in mechanical performance.

**Figure 10:**
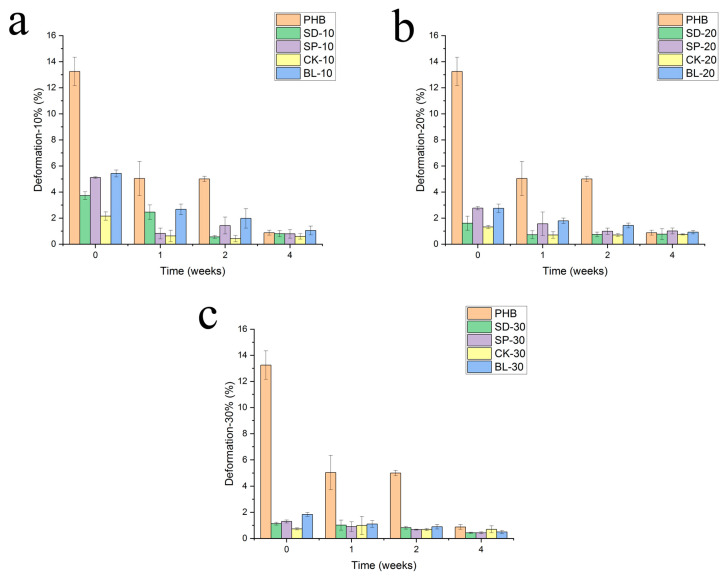
Evolution of the deformation at the break of composites with the exposure time in Xenotest chamber. Reinforcement: (**a**) 10%, (**b**) 20% and (**c**) 30%.

Figure 10 shows the profiles of elongation at break. Comparatively, in terms of the capacity for deformation (elongation at break), an important decrease has been obtained for plain PHB. This decrease is related to the degradation process experienced by PHB [6]. The elongation at break decreased from 13–14% when the material had not been exposed to the specific environmental conditioning to less than 1% after 28 days, this being the highest loss of this property during the first week of exposure. The composites reinforced with fibres also lost deformability until reaching values close to that of PHB, but relatively, this loss is lower as the composite intrinsically has a lower deformation because of the presence of a rigid phase in the composite. In any case, exposure to the radiation and humidity leads to a weak material explained by the degradation process of PHB.

Turning to the statistical test, as in the previous studies of contact angle and water uptake, the individual average values with a high value of standard deviation showed nonstatistical differences compared with the previous and the posterior average value in the trend. In the particular case of the statistical study of the profiles of elongation at break, due to the low value of the property caused by the degradation of the material after exposure, no statistical differences were obtained for any of the composites. That is the case for the values after 2 and 4 weeks, where no differences were obtained at a 95% interval of confidence.

## 4. Conclusions

In this work, the effect of the incorporation of barley fibres—preconditioned by different physical and chemical procedures—to PHB have been studied. 

After exposure to controlled environmental conditions, a qualitative degradative process has been observed in all the studied samples, visually identified by the bleaching of the surface of the samples. The expected degradation process, together with the low compatibility between the components of the composite, also influenced the water uptake capacity and contact angle of the materials. The water uptake of the samples increased with the exposure time due to the greater capacity for water diffusion after degradation. In the case of contact angle, it decreased due to the presence of oxidated polar compounds.

A better performance of mechanical properties after the environmental treatment has been observed for composites reinforced with chemically modified fibres (CK and BL) compared to those reinforced with physically treated (SD and SP). The improvement is related to the increase of cellulose content in these fibres by the removal of lignin (cooking and bleaching processes) which led to a higher reinforcement effect in the composites.

With all of these arguments, and due to the low requirements for mechanical performance of the materials needed for the application, it is considered that the use of lignocellulosic fibres, without any chemical treatment, allows the obtaining of materials resistant enough to keep their properties over several weeks, finishing their working lives after direct disintegration in the crop field.

## Figures and Tables

**Table 1:** Routine of chamber conditioning to simulate working environment. Rain: 500 mL/min of distilled water delivered on the top of the samples. *: temperature controlled by the rain (distilled water at room temperature). **: no constant temperature due to no radiation or heating period.

Phase	Starting Time (min)	Ending Time (min)	Radiation Power (W·m^−2^)	Temperature (°C)	Relative Humidity (%)	Rain (Yes/No)
1	0	101	42	63	50	no
2	102	119	42	--*	50	yes
3	120	159	0	--**	95	no

## Data Availability

Not applicable.
